# From dysbiosis to prediction: a novel gut microbiota–derived index for spontaneous bacterial peritonitis in HBV-related cirrhosis

**DOI:** 10.3389/fimmu.2026.1753063

**Published:** 2026-02-16

**Authors:** Zhewen Zhou, Xiu Sun, Danying Cheng, Huichun Xing

**Affiliations:** 1 Center of Liver Diseases Division 3, Beijing Ditan Hospital, Capital Medical University, Beijing, China; 2 National Center For Infectious Diseases, Beijing, China; 3 Peking University Ditan Teaching Hospital, Beijing, China

**Keywords:** 16S rRNA, gut microbiota–derived index, HBV-related cirrhosis, prediction model, spontaneous bacterial peritonitis

## Abstract

**Objective:**

Spontaneous bacterial peritonitis (SBP) is a severe complication of hepatitis B virus (HBV)-related cirrhosis, in which gut microbiota dysbiosis plays a pivotal role. This study aimed to characterize microbial alterations, establish a microbiota-derived index (SBP-MI), evaluate longitudinal changes, and develop an SBP risk prediction model (SBP-RP).

**Methods:**

A total of 135 participants were included: healthy controls (n=40), compensated cirrhosis (n=30), cirrhosis with ascites but without SBP (n=40), and SBP (n=25). Fecal 16S rRNA sequencing and clinical data were obtained. An additional 140 cirrhotic patients with ascites were prospectively followed for 6 months, with SBP occurrence as the endpoint; 40 provided paired fecal samples. SBP-MI was constructed from key microbial shifts, and multivariable Firth logistic regression identified independent predictors.

**Results:**

SBP was characterized by enrichment of pathogenic taxa (*Escherichia–Shigella, Klebsiella, Veillonella, Streptococcus*) and depletion of short-chain fatty acid producers (*Prevotella, Roseburia, Faecalibacterium, Bacteroides*), forming the basis of SBP-MI. During follow-up, improved patients had greater microbial diversity and beneficial commensals, whereas progression was linked to *Haemophilus* expansion. SBP-MI effectively tracked these changes and outperformed the Hepatitis B Cirrhosis Dysbiosis Index. Multivariable analysis identified INR, previous history of SBP, and SBP-MI as independent predictors. The SBP-RP yielded an AUC of 0.91(95% CI: 0.82–0.99), with calibration that appeared acceptable in this cohort.

**Conclusion:**

Distinct dysbiosis characterizes SBP. SBP-MI captures microbial imbalance and progression, while the SBP-RP model integrating microbial and clinical factors provides promising predictive value for early risk stratification.

## Graphical Abstract

**Figure:**
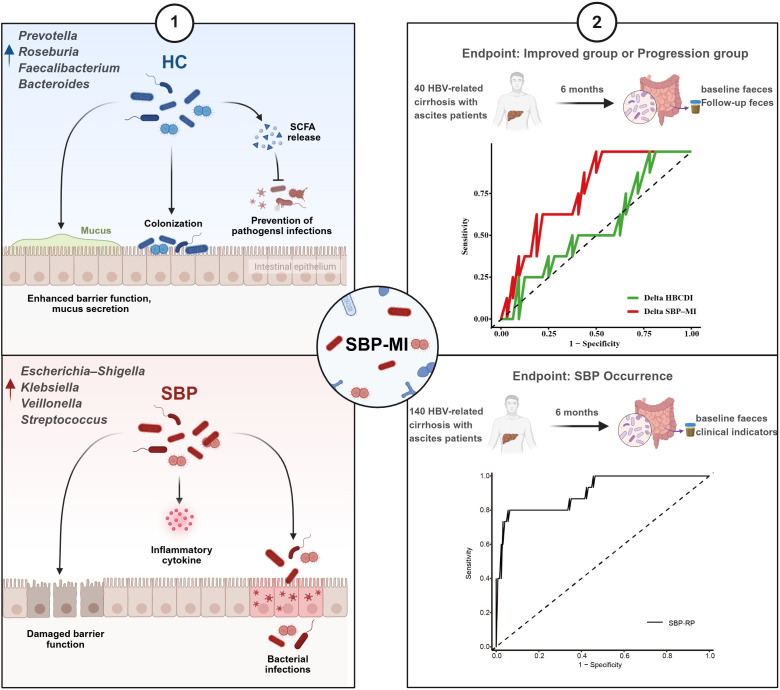

## Highlights

- We developed a spontaneous bacterial peritonitis microbiota-derived index (SBP-MI) that captures gut microbial imbalance in spontaneous bacterial peritonitis.
- In patients with hepatitis B virus–related cirrhosis and ascites, SBP-MI reflects both disease improvement and progression.
- By integrating SBP-MI with routine clinical variables, we constructed a spontaneous bacterial peritonitis risk prediction model (SBP-RP) to stratify SBP risk.

## 1 Introduction

Cirrhosis is a major cause of mortality worldwide (1). Spontaneous bacterial peritonitis (SBP) is one of the most common and severe complications of cirrhosis, usually occurring in end-stage disease. Among hospitalized cirrhotic patients with ascites, the 1-year recurrence rate of SBP is as high as 40%–70% (2). However, despite the availability of highly sensitive detection techniques, the positive culture rate remains low (3), and a subset of SBP patients presents without typical clinical symptoms. Hepatitis B virus (HBV)-related cirrhosis with ascites represents a high -risk state for spontaneous bacterial peritonitis, in which alterations of the gut microbiota appear to play an important role. Nevertheless, current evidence remains limited. Therefore, identifying high-risk patients early using microbiome-informed markers and implementing timely preventive strategies are critical to reducing the incidence and recurrence of SBP.

The pathogenesis of spontaneous bacterial peritonitis in cirrhosis is complex and is closely linked to intestinal mucosal barrier dysfunction and bacterial translocation (4). Causative pathogens predominantly originate from intestinal commensals, and ascitic fluid cultures frequently yield Gram-negative bacteria such as *Escherichia coli*, *Streptococcus*, *Enterococcus*, and *Klebsiella* species (3). Several microbiota-based indices have been proposed to quantify dysbiosis in cirrhosis, including the cirrhosis dysbiosis ratio (CDR) (5), the probability of disease score (POD) (6), and the hepatitis B cirrhosis dysbiosis indicator (HBCDI) (7). Although simple to calculate, the CDR was not developed in HBV-related cirrhosis and has limited applicability. The POD index has been reported to predict SBP. However, its algorithmic complexity and limited consideration of etiology, together with the predominance of cross-sectional sampling, make it difficult to evaluate microbiota dynamics during disease progression. The HBCDI was derived in HBV-related cirrhosis, but its value for SBP risk assessment and for serial monitoring has not been established. Overall, existing indices do not provide a simple measure that both discriminates SBP risk and reflects disease evolution in HBV-related cirrhosis with ascites.

Therefore, the present study aimed to systematically characterize the gut microbiota in HBV-related cirrhosis across different disease stages, to develop the SBP microbiota-derived index (SBP-MI) as a novel dysbiosis index, and to evaluate its potential in longitudinal follow-up. Furthermore, we integrated SBP-MI with clinical factors to establish a risk prediction model for SBP. This microbiome-informed framework may help identify patients at higher SBP risk and support targeted preventive management.

## 2 Materials and methods

### 2.1 Study design and participants

**Figure 1:**
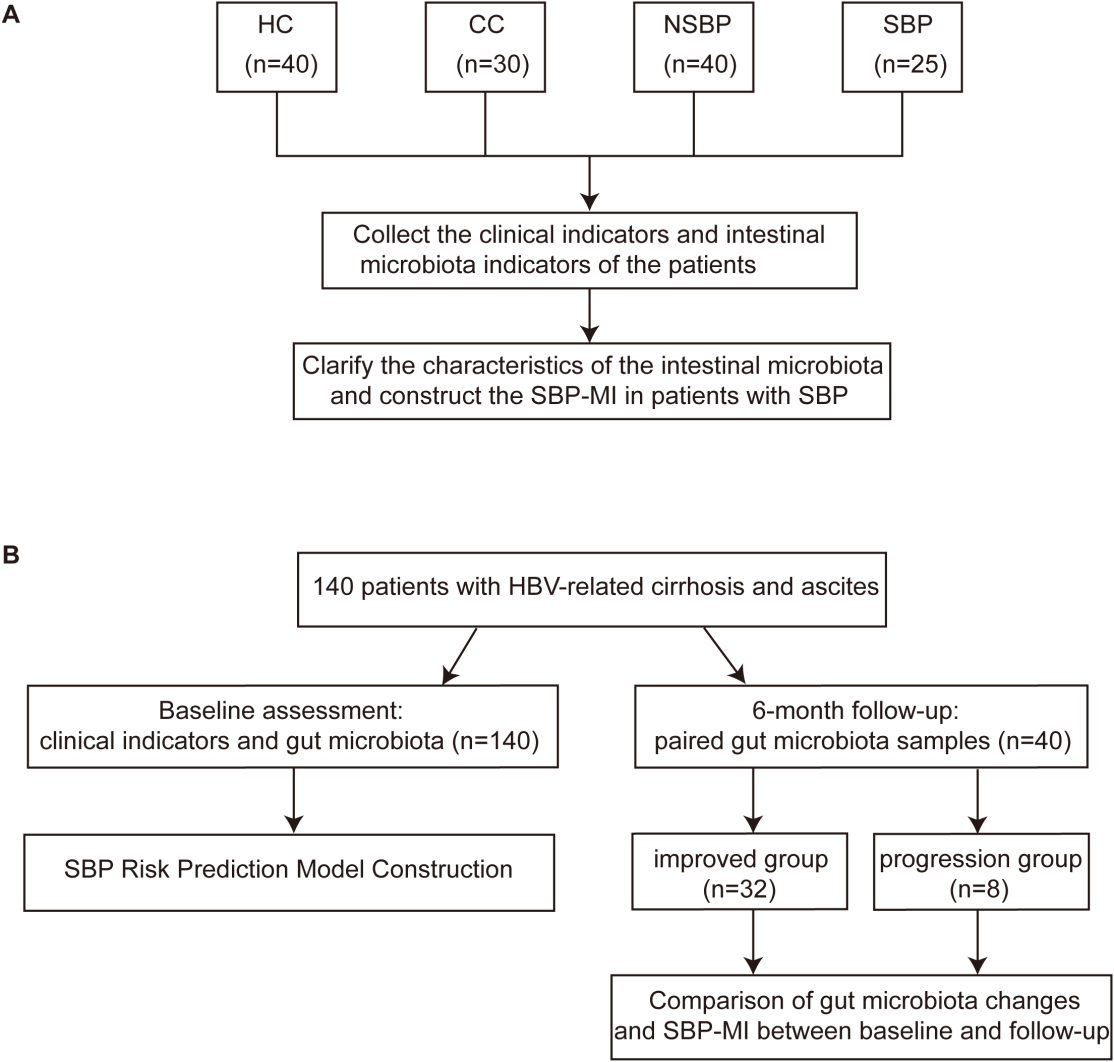
Design of the case-control study and the prospective cohort. **(A)** Case-control study comparing clinical parameters and gut microbiota among HC (n=40), CC (n=30), NSBP (n=40), and SBP (n=25), with construction of the SBP-MI. **(B)** A total of 140 patients with HBV-related cirrhosis and ascites were enrolled and underwent baseline clinical assessment and gut microbiota profiling (n = 140) for construction of the SBP risk prediction model, with incident SBP as the primary endpoint. Forty patients provided paired fecal samples at baseline and 6-month follow-up, including an improved group (n = 32) and a progression group (n = 8), to assess longitudinal changes in gut microbiota composition and SBP-MI between time points. HC, healthy control; CC, compensated HBV-related cirrhosis; NSBP, HBV-related cirrhosis with ascites but without spontaneous bacterial peritonitis; SBP, HBV-related cirrhosis with spontaneous bacterial peritonitis; SBP-MI, Spontaneous bacterial peritonitis microbiota-derived index.

This study was conducted at Beijing Ditan Hospital, Capital Medical University, between September 2019 and November 2021, and included both a case-control study and a prospective cohort component (**Figure 1**). The protocol was approved by the institutional ethics committee (DT-IRB-2018-040-01), and written informed consent was obtained. The prospective cohort was registered in the Chinese Clinical Trial Registry (trial registration number: ChiCTR-ROC-17013343).

In the case-control study, four groups were enrolled: healthy controls (HC, n=40), compensated HBV-related cirrhosis (CC, n=30), cirrhosis with ascites but without SBP (NSBP, n=40), and cirrhosis with SBP (SBP, n=25). Clinical and microbial characteristics were analyzed, and the SBP-MI was constructed from differential taxa and evaluated for discrimination. We conducted an unmatched, stratified case-control study with consecutive enrollment under prespecified criteria. The resulting group sizes and age distributions reflect real-world accrual patterns and underlying disease severity.

In the prospective cohort, 140 patients with HBV-related cirrhosis and ascites were followed for 6 months, with SBP occurrence as the primary endpoint. Stool samples were collected from 140 patients at baseline. The prospective cohort enrolled ascitic patients without SBP at baseline. Accordingly, SBP cases included in the case-control study were not part of the baseline prospective cohort. The SBP risk prediction model (SBP-RP) was constructed using baseline SBP-MI and baseline clinical variables. A subset of 40 patients provided paired baseline and follow-up fecal samples, enabling assessment of clinical progression or improvement on the basis of microbial dynamics and changes in SBP-MI.

### 2.2 Inclusion and exclusion criteria

Exclusion criteria: The case-control and prospective studies applied identical exclusion criteria. Exclusion criteria included non-HBV etiologies of liver disease, hepatocellular carcinoma, other malignancies, severe comorbidities, active tuberculosis, concomitant extrahepatic infections, non-hepatic ascites, and the presence of a transjugular intrahepatic portosystemic shunt (TIPS) at baseline and follow-up.

Inclusion criteria: Core inclusion criteria were harmonized in the case-control and prospective studies. Core inclusion criteria were >18 years of age and had not received antibiotics, probiotics, proton pump inhibitors, bowel-preparation cathartics, or motility-altering agents including lactulose within 1 month before enrollment. This 1-month window was used to reduce recent medication-related perturbations of the gut microbiota at the time of sampling. Participants had not consumed yogurt and had not used other microbiota-altering agents. All participants consumed a mixed diet of plant- and animal-based foods, without vegetarian or other restrictive dietary patterns. Because the enrolled clinical states differed, disease-specific inclusion criteria were as follows. In the case-control study: HC required normal clinical, laboratory, and ultrasound findings without major systemic disease. CC was defined by chronic HBV infection with histologic, imaging, or endoscopic evidence of cirrhosis, liver stiffness >12.0 kPa, and Child–Pugh A (<7). NSBP required chronic HBV infection, cirrhosis with ascites, and absence of infection, encephalopathy, or gastrointestinal bleeding. SBP required fulfillment of NSBP criteria plus acute peritonitis or systemic inflammatory response, and an ascitic polymorphonuclear cell count ≥0.25 × 10^9^/L without secondary causes. In the prospective cohort study: HBV-related cirrhosis with ascites but without SBP at baseline.

### 2.3 Definition of improved and progression groups

Patients with HBV-related cirrhosis and ascites were prospectively followed for 6 months.

Progression group: Progression was defined within 6 months if any of the following applied:

1. Ascites grade increased from baseline according to EASL guidelines (8). Grade 1 is mild and detectable only by ultrasound. Grade 2 is moderate with symmetrical abdominal distension. Grade 3 is large or tense with marked abdominal distension.
2. Spontaneous bacterial peritonitis, death, or liver transplantation occurred.
3. Biochemical worsening was present, defined as ALT or AST increasing by at least 50% from baseline and exceeding the upper limit of normal, defined as ALT greater than 40 U/L or AST greater than 50 U/L, or serum albumin decreasing from baseline or falling below 40 g/L.

Improved group: Improved status required that all of the following criteria were met:

1. Ascites grade decreased or remained unchanged.
2. No spontaneous bacterial peritonitis, death, or liver transplantation.
3. Biochemical improvement, defined as decreases in ALT and AST from baseline or values within the normal range (ALT ≤ 40 U/L; AST ≤ 50 U/L), together with an increase in serum albumin from baseline or restoration to the normal range (40–55 g/L).

Patients with stable disease who had ALT ≤ 40 U/L and AST ≤ 50 U/L and albumin 40 to 55 g/L at baseline, remained within these ranges at follow-up, and had no increase in ascites grade and no SBP, death, or liver transplantation were classified as Improved.

### 2.4 Sample collection and sequencing

Demographic and clinical data were recorded at enrollment. Assessments of oesophago-gastric varices and measurement of portal pressure by the hepatic venous pressure gradient (HVPG) are invasive and are not routinely indicated outside the prophylaxis or treatment of variceal bleeding. Transient elastography may be unreliable in the presence of tense ascites or acute infection. In SBP, clinical instability at enrollment therefore precluded routine assessment of oesophago-gastric varices, portal pressure, and transient elastography. Fasting peripheral blood samples were collected for biochemical and virological assays. Fresh stool samples were obtained using sterile containers, transported on ice, aliquoted within 30 minutes, and stored at –80 °C until analysis. Microbial DNA extracted from fecal samples was used to amplify the V3–V4 region of the 16S rRNA gene, and the resulting libraries were sequenced on the Illumina NovaSeq platform. Further details are provided in the **Supplementary Methods**.

### 2.5 Construction of the SBP-MI

Taxa for the SBP-MI were selected in the case-control discovery set (HC, CC, NSBP, SBP) using two complementary genus level criteria. First, LEfSe was used to determine enrichment direction and effect size, with LDA scores defining the candidate pool. Second, four group mean relative abundance heatmaps were inspected to confirm trends consistent with disease staging. Detailed genus selection was described in the Results section. The SBP-MI was then calculated as follows: *x_g_* denotes the genus-level relative abundance in each sample. A pseudocount of 10^–6^ was added to each *x_g_* to handle zero values and enable log-ratio calculation.


$$\text{SBP}-\text{MI}=log10(\frac{\sum_{\left(g\in \{Streptococcus,Escherichia-Shigella,Klebsiella,Veillonella\})(x_{g}+10^{-6}\right)}}{\sum_{\left(g\in \{Prevotella,Roseburia,Faecalibacterium,Bacteroides\})(x_{g}+10^{-6}\right)}})$$

HBCDI was previously developed by our group to quantify gut microbial imbalance in patients with HBV-related cirrhosis. The HBCDI was then calculated as follows: HBCDI = (*Escherichia − Shigella* + *Streptococcus* + *Lactobacillus)/(Ruminococcus* + *Prevotella* + *Bacteroides*) (7, 9).

### 2.6 Bioinformatics and statistical analysis

Raw reads were processed in QIIME 2 (release 2025.7) for 97% OTU clustering, taxonomic assignment, and diversity analyses. Differential taxa were identified using LEfSe after exporting the feature table and metadata. Baseline laboratory data were complete, with no missing values. Data visualization was performed in R (v4.5.1). Group comparisons were conducted with appropriate parametric or non-parametric tests, and categorical variables with χ² or Fisher’s exact test. Given the limited number of SBP events in the prospective cohort, predictive modeling was performed using Firth’s penalized logistic regression to reduce small-sample bias and address potential separation. Model performance was assessed by ROC analysis, bootstrap validation, and calibration. P<0.05 (two-sided) was considered significant. Details are provided in the **Supplementary Methods**.

## 3 Results

### 3.1 Baseline clinical characteristics of HC, CC, NSBP, and SBP

A total of 30 patients with CC, 40 with NSBP, 25 with SBP, and 40 HC were included in the analysis. Baseline characteristics of the four groups are summarized in **Table 1**. Compared with HC and CC, NSBP and SBP were characterized by significantly higher TBil and lower RBC, ALB, HGB, and PLT (P < 0.05). The median NE% was numerically higher in the SBP group; however, the difference was not statistically significant. Smoking status was comparable across groups. Diuretic therapy was universal in the NSBP and SBP cohorts, consistent with standard ascites management. Model for End-Stage Liver Disease (MELD) increased progressively with disease stage, and Child–Pugh class shifted toward more advanced categories. In the SBP group, 5 of 25 patients (20%) had a prior history of SBP.

**Table 1:** Baseline clinical characteristics of HC, CC, NSBP, and SBP.

Characteristics	HC (n=40)	CC (n=30)	NSBP (n=40)	SBP (n=25)
Male (%)	30 (75.0)^a^	17 (56.7)^a^	27 (67.5)^a^	19 (76.0)^a^
BMI	23.6 (22.5–25.2)^a^	24.0 (22.9–24.7)^a^	24.0 (23.0–25.3)^a^	23.1 (22.1–25.0)^a^
Age	52.5 ± 7.6^a^	49.2 ± 9.3^a^	52.5 ± 9.4^a^	60.6 ± 12.5^b^
ALT (U/L)	17.4 (13.0–22.1)^a^	24.2 (21.1–32.0)^b^	27.2 (16.1–37.3)^b^	19.5 (14.0–32.7)a^b^
AST (U/L)	18.8 (17.2–21.6)^a^	26.6 (23.2–34.3)^b^	34.0 (25.3–50.1)^b^	27.2 (19.9–59.3)^b^
ALB (g/L)	46.4 (44.3–47.4)^a^	44.3 (39.4–48.3)^a^	32.0 (27.8–37.5)^b^	29.5 (26.3–32.8)^b^
TBil (μmol/L)	13.8 (11.8–15.2)^a^	14.9 (11.4–22.4)^a^	30.1 (18.2–53.8)^b^	51.6 (31.9–74.0)^b^
Cr (μmol/L)	73.5 (63.1–80.4)^a^	65.2 (57.6–72.2)^a^	66.9 (60.4–76.1)^a^	77.3 (60.5–88.1)^a^
WBC (10^9^/L)	5.8 (4.8–6.1)^a^	4.3 (3.7–5.4)^b^	2.9 (2.2–3.6)^c^	3.0 (2.4–4.7)^c^
NE (%)	56.2 (52.3–61.5)^a^	56.1 (53.9–61.0)^a^	57.1 (50.6–65.1)^a^	70.1 (50.6–75.7)^a^
RBC (10^12^/L)	4.8 (4.5–5.0)^a^	4.8 (4.3–5.0)^a^	3.5 (3.2–4.2)^b^	3.1 (2.6–3.9)^b^
HGB (g/L)	146.0 (140.0–155.0)^a^	143.0 (129.8–156.8)^a^	114.5 (96.8–127.2)^b^	104.0 (83.5–111.0)^b^
PLT (10^9^/L)	235.5 (180.8–264.2)^a^	104.0 (92.0–135.8)^b^	56.0 (42.4–81.2)^c^	62.0 (41.0–88.2)^c^
Dietary habit	Mix	Mix	Mix	Mix
Smoking (%)	8(20)^a^	7(23.3)^a^	7(17.5)^a^	5(20)^a^
Diuretics (%)	0(0)^a^	0(0)^a^	40(100)^b^	25(100)^b^
Previous history of SBP (%)	0(0)^a^	0(0)^a^	0(0)^a^	5(20)^b^
MELD	7.0(7.0-7.2)^a^	8.5(7.0-9.8)^b^	12.0(9.8-15.0)^c^	19.0(15.0-21.0)^d^
Child-Pugh A, n (%)	40 (100)^a^	30 (100)^a^	12 (30)^b^	1 (4)^c^
Child-Pugh B, n (%)	0 (0)^a^	0 (0)^a^	21 (52.5)^b^	10 (40)^c^
Child-Pugh C, n (%)	0 (0)^a^	0 (0)^a^	7 (17.5)^b^	14 (56)^c^

Data were shown as mean ± SD, median (IQR), or n (%). Different superscript letters (a, b, c, d) denote significant differences between groups (P < 0.05).

HC, Healthy control; CC, Compensated HBV-related cirrhosis; NSBP, HBV-related cirrhosis with ascites but without spontaneous bacterial peritonitis; SBP, HBV-related cirrhosis with spontaneous bacterial peritonitis; BMI, body mass index; ALT, alanine aminotransferase; AST, aspartate aminotransferase; ALB, albumin; TBil, total bilirubin; Cr, creatinine; WBC, white blood cell; NE, neutrophil percentage; RBC, red blood cell; HGB, hemoglobin; PLT, platelet; MELD, Model for End-Stage Liver Disease.

### 3.2 Gut microbiota dysbiosis is most pronounced in SBP

**Figure 2:**
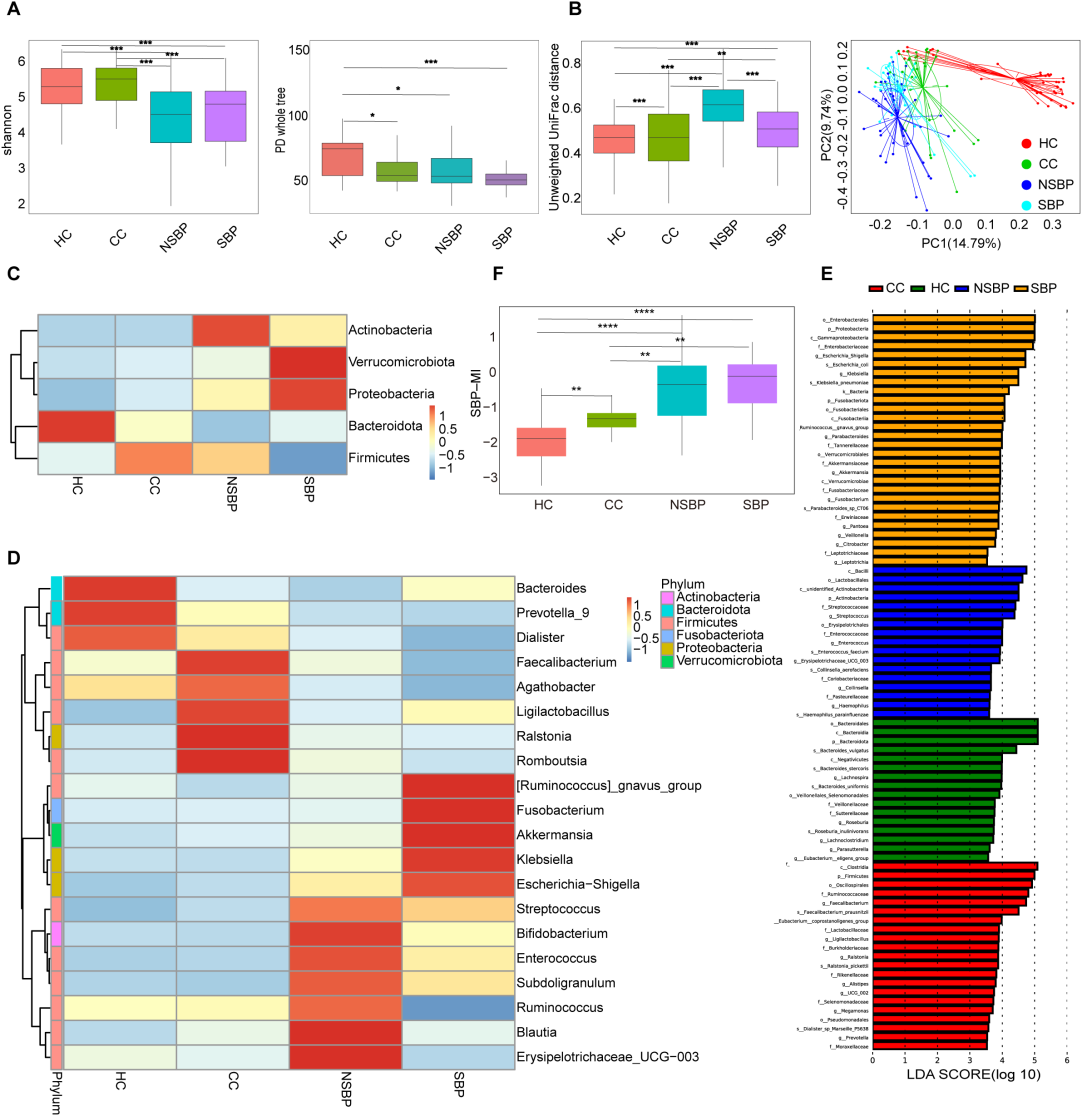
Gut microbial diversity and composition in HC, CC, NSBP, and SBP. **(A)** α-diversity indices (Shannon and PD whole tree); **(B)** β-diversity based on Unweighted UniFrac distances and PCoA; **(C)** Relative abundance at the phylum level; **(D)** Relative abundance at the genus level; **(E)** Differential taxa identified by LEfSe analysis(LDA>3.5); **(F)** Boxplots showing the distribution of SBP-MI among HC, CC, NSBP, and SBP groups. HC, Healthy control; CC, Compensated HBV-related cirrhosis; NSBP, HBV-related cirrhosis with ascites but without spontaneous bacterial peritonitis; SBP, HBV-related cirrhosis with spontaneous bacterial peritonitis; PCoA, principal coordinates analysis; LEfSe, linear discriminant analysis effect size; LDA, linear discriminant analysis; SBP-MI, Spontaneous bacterial peritonitis microbiota-derived index. *P < 0.05, **P < 0.01, ***P < 0.001, and ****P < 0.0001.

Analysis of α-diversity showed that the HC group had the highest Shannon and phylogenetic diversity (PD) whole-tree indices, indicating the greatest microbial and phylogenetic diversity. Compared with HC and CC, both NSBP and SBP exhibited significantly decreased diversity (P < 0.05), with SBP showing the lowest PD whole-tree index (**Figure 2A**). Based on unweighted UniFrac β-diversity (**Figure 2B**), distance values in NSBP and SBP were higher than those in HC and CC. Principal coordinates analysis (PCoA) further revealed a clear separation between diseased groups and healthy controls, suggesting marked alterations in community composition during disease progression.

At the phylum level (**Figure 2C**), *Firmicutes*, *Bacteroidota*, *Proteobacteria*, *Actinobacteria*, and *Verrucomicrobiota* together accounted for more than 90% of the total abundance. HC was dominated by *Bacteroidota*. With disease progression, *Bacteroidota* progressively decreased, whereas *Proteobacteria* markedly increased. In SBP, *Firmicutes* were reduced, while *Proteobacteria* and *Verrucomicrobiota* were enriched. At the genus level (**Figure 2D**), HC was enriched in *Bacteroides*, *Prevotella_9*, and *Faecalibacterium*, whereas pathogenic genera such as *Escherichia–Shigella*, *Klebsiella*, and *Streptococcus* peaked in SBP. LEfSe analysis identified stage-specific enriched taxa (**Figure 2E**): *Roseburia* in HC; *Faecalibacterium* and *Prevotella* in CC; *Enterococcus* and *Haemophilus* in NSBP; and *Escherichia–Shigella*, *Klebsiella*, and *Veillonella* in SBP. Collectively, these findings indicate that the progression of HBV-related cirrhosis is accompanied by a gradual loss of commensals and an expansion of potential pathogens, with the shift being most pronounced at the SBP stage.

### 3.3 Construction of the SBP-MI index

To quantify gut microbial imbalance associated with disease progression, we constructed the SBP-MI by integrating the results of LEfSe analysis and genus-level abundance heatmaps. Accordingly, the numerator comprised *Escherichia–Shigella*, *Klebsiella*, and *Veillonella*, which were enriched in SBP with LDA scores of 4.71, 4.49, and 3.80, respectively. *Streptococcus* showed a stepwise increase from HC/CC to NSBP/SBP on the heatmap (**Figure 2D**) and was included to capture the progression toward SBP. The denominator comprised *Roseburia*, *Bacteroides*, *Prevotella*, and *Faecalibacterium*. *Roseburia* was enriched in HC (LDA 3.74). *Bacteroides* and *Prevotella* showed the highest mean relative abundance in HC and were clearly separated from the other groups. *Faecalibacterium* was higher in HC and CC and lowest in SBP. The SBP-MI differed significantly across the four groups (P < 0.001). Pairwise comparisons revealed significant differences between HC and NSBP, HC and SBP, as well as between CC and SBP (**Figure 2F**). Trend analysis further demonstrated that the SBP-MI increased progressively with disease progression (P < 0.001). In cirrhosis (CC, NSBP, SBP), SBP-MI correlated positively with MELD (Spearman ρ=0.36; 95% CI, 0.17–0.54; P = 0.0003).

### 3.4 Gut microbial shifts associated with clinical improvement and progression in ascites

We prospectively enrolled 140 patients with HBV related cirrhosis and ascites and followed them for 6 months. Forty patients provided paired fecal samples at baseline and follow-up. Of these, 32 were classified as improved and 8 as progressed. The paired-sample analyses were designed to determine whether SBP-MI and overall community composition track clinical improvement versus progression.

**Figure 3:**
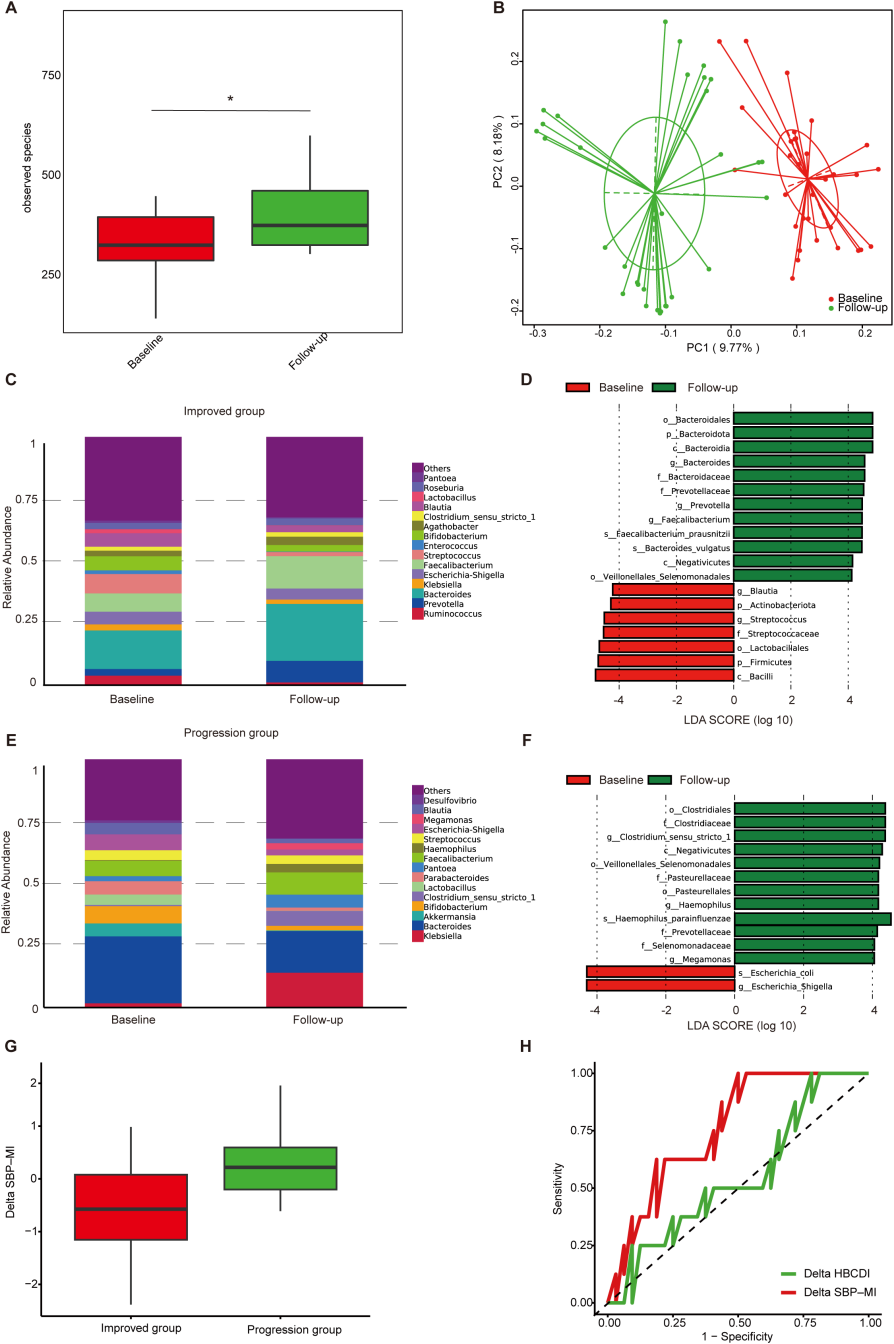
Gut microbiota dynamics in ascites patients during follow-up. **(A)** α-diversity based on observed species in the improved group; **(B)** PCoA based on Binary Jaccard distance in the improved group; **(C)** Genus-level relative abundance in the improved group; **(D)** Differential taxa identified by LEfSe analysis in the improved group (LDA>4); **(E)** Genus-level relative abundance in the progression group; **(F)** Differential taxa identified by LEfSe analysis in the progression group (LDA>4); **(G)** Boxplots of Delta SBP-MI in the progression and improved groups; **(H)** ROC curve analysis of Delta SBP-MI (AUC 0.76, 95%CI 0.60–0.93) and Delta HBCDI (AUC 0.55, 95%CI 0.33–0.78) for predicting clinical outcomes. PCoA, principal coordinates analysis; LEfSe, linear discriminant analysis effect size; LDA, linear discriminant analysis; ROC, receiver operating characteristic; SBP-MI, Spontaneous bacterial peritonitis microbiota-derived index; HBCDI, Hepatitis B Cirrhosis Dysbiosis Indicator. *P < 0.05.

In the improved group (**Supplementary Table S1**), ALB, HGB, and RBC increased, whereas TBil, AFP, HBV DNA, and MELD decreased at follow-up (P < 0.05). α-diversity based on observed species was significantly higher at follow-up (**Figure 3A**), and PCoA revealed separation between baseline and follow-up samples (**Figure 3B**). At the genus level, *Klebsiella*, *Streptococcus*, and *Enterococcus* decreased (**Figure 3C**). LEfSe confirmed reduced *Streptococcus* and enrichment of *Bacteroides*, *Prevotella*, and *Faecalibacterium* (**Figure 3D**).

In the progression group (**Supplementary Table S2**), TBil and MELD increased at follow-up (P < 0.05). At the genus level, *Klebsiella* and *Haemophilus* increased, whereas *Akkermansia*, *Bifidobacterium*, and *Lactobacillus* decreased (**Figure 3E**). LEfSe revealed higher abundance of *Escherichia–Shigella* at baseline and *Haemophilus parainfluenzae* at follow-up (**Figure 3F**). Overall, the improved group was associated with increased diversity and enrichment of commensals, whereas the progression group was characterized by persistence or further enrichment of potential pathogens.

To further assess longitudinal changes in microbial indices, we calculated ΔSBP-MI and ΔHBCDI as the differences between follow-up and baseline values in the 40 patients with paired fecal samples. (ΔSBP-MI=SBP-MI_follow-up_-SBP-MI_baseline_; ΔHBCDI= HBCDI_follow-up_- HBCDI_baseline_) ΔSBP-MI was significantly higher in the progression group than in the improved group (P = 0.02) (**Figure 3G**). ROC analysis showed that ΔSBP-MI had an AUC of 0.76 (95% CI: 0.60–0.93). At the optimal cut-off of −0.62, ΔSBP-MI achieved a sensitivity of 1.00 and a specificity of 0.50. By comparison, ΔHBCDI showed lower discrimination, with an AUC of 0.55 (95% CI: 0.33–0.78) (**Figure 3H**), supporting the better performance of ΔSBP-MI in distinguishing improved from progressed outcomes.

### 3.5 Development of the SBP-RP model based on clinical and microbial factors

**Figure 4:**
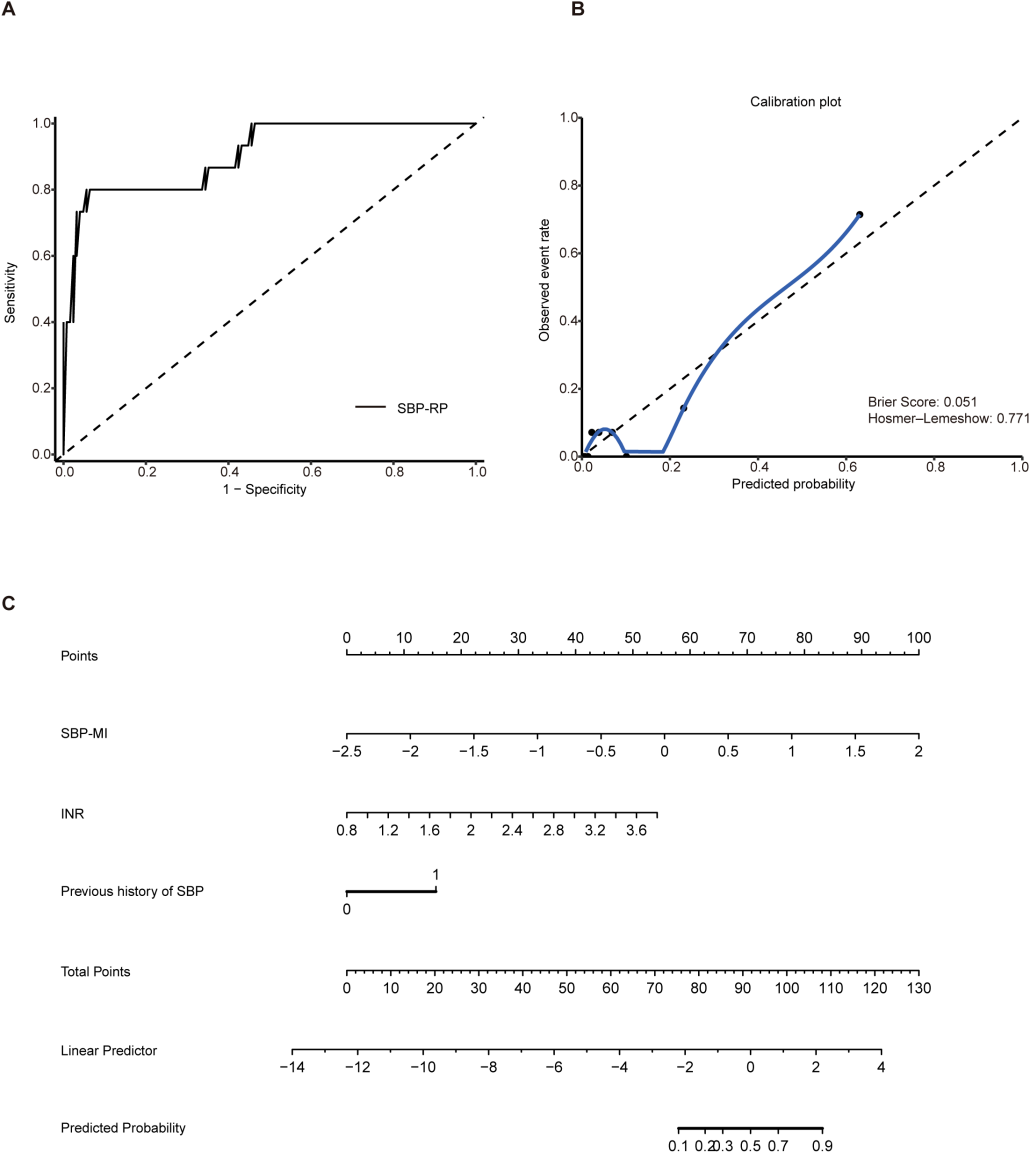
Construction of the SBP-RP model. **(A)** ROC curve of SBP-RP (AUC 0.91, 95% CI: 0.82–0.99); **(B)** Calibration plot of the SBP-RP model; **(C)** Nomogram of SBP-RP. SBP, spontaneous bacterial peritonitis; SBP-RP, SBP risk prediction model; ROC, receiver operating characteristic; AUC, area under the curve.

In a 6-month prospective cohort comprising 140 patients with HBV-related cirrhosis and ascites, the primary endpoint was incident SBP, which occurred in 15 patients (10.7%). No patient was lost to follow-up, died, or underwent liver transplantation. Baseline clinical characteristics of patients with HBV-related cirrhosis and ascites were summarized in **Supplementary Table S3**. The association between a previous history of SBP and incident SBP during follow-up was summarized in **Supplementary Table S4**. The AUC for SBP-MI was 0.83 (95% CI: 0.74–0.92), compared with 0.73 for CDR (95% CI: 0.58–0.89) and 0.71 for HBCDI (95% CI: 0.57–0.85), indicating superior discriminatory performance of SBP-MI (**Supplementary Figure S1**). As shown in **Table 2**, 10 variables with P < 0.10 in univariable analyses were entered into the multivariable Firth logistic regression. SBP-MI, INR, and previous history of SBP remained independent predictors (P < 0.05). The final predictive equation was: SBP-RP = –6.8107 + 2.9873 × SBP-MI + 2.4316 × INR + 2.0950 × previous history of SBP. Discrimination was good, with an AUC of 0.91 (95% CI: 0.82–0.99). At the optimal cutoff of 0.262, the sensitivity was 0.80 and the specificity was 0.944 (**Figure 4A**). Bootstrap optimism correction (500 resamples; seed 2025) gave an optimism-corrected AUC of 0.90 (95% CI: 0.82–0.99). Calibration was acceptable overall. The calibration slope was 1.11 (95% CI: 1.08–1.30) and the Brier score was 0.051, with a non-significant Hosmer–Lemeshow test (P = 0.77). However, the calibration curve deviated from the ideal line at higher predicted probabilities (**Figure 4B**). These findings should be interpreted cautiously given the low number of events. A nomogram was generated to enhance clinical applicability (**Figure 4C**). These results suggest that the SBP-RP model can stratify short-term SBP risk in HBV related cirrhosis with ascites. Its longer-term performance will be assessed with extended follow-up and validated in larger external cohorts.

**Table 2:** Univariable and multivariable logistic regression analysis of risk factors for SBP.

Characteristics	Univariable		Multivariable	
	OR (95% CI)	p value	OR (95%CI)	p value
SBP-MI	10.953 (3.616 – 44.297)	<0.001	19.832 (5.091 – 140.054)	<0.001
INR	3.950 (1.483 – 11.296)	0.007	11.377 (2.989 – 55.277)	<0.001
HBCDI	1.153 (1.049 – 1.394)	0.008	–	–
NE	1.048 (1.006 – 1.097)	0.018	–	–
ALB	0.904 (0.821 – 0.986)	0.021	–	–
CHE	1.000 (0.999 – 1.000)	0.022	–	–
Na	0.838 (0.729 – 0.962)	0.029	–	–
Previous history of SBP	3.019 (1.016 – 8.855)	0.057	8.125 (1.976 – 40.901)	0.003
Cr	1.005 (0.995 – 1.013)	0.070	–	–
Age	1.043 (0.999 – 1.090)	0.083	–	–
TBil	1.007 (0.998 – 1.015)	0.158	–	–
Gender	2.558 (0.729 – 13.475)	0.231	–	–
RBC	0.658 (0.312 – 1.377)	0.251	–	–
WBC	1.123 (0.838 – 1.455)	0.666	–	–
PLT	1.002 (0.991 – 1.010)	0.688	–	–
Diabetes	1.469 (0.412 – 4.507)	0.739	–	–
ALT	1.001 (0.993 – 1.006)	0.788	–	–
AST	1.001 (0.993 – 1.006)	0.808	–	–
log10HBV DNA	1.027 (0.767 – 1.317)	0.873	–	–
GLU	1.043 (0.805 – 1.255)	0.997	–	–

SBP, spontaneous bacterial peritonitis; SBP-MI, Spontaneous bacterial peritonitis microbiota-derived index; INR, international normalized ratio; HBCDI, Hepatitis B Cirrhosis Dysbiosis Indicator; NE, neutrophil percentage; ALB, albumin; CHE, cholinesterase; Cr, creatinine; TBil, total bilirubin; RBC, red blood cell; WBC, white blood cell; PLT, platelet; ALT, alanine aminotransferase; AST, aspartate aminotransferase; HBV DNA, hepatitis B virus DNA; GLU, glucose.

## 4 Discussion

SBP represents a serious complication of cirrhosis and is strongly linked to gut dysbiosis and bacterial translocation (4). Although the role of microbiota in cirrhosis has been increasingly recognized, microbiota-derived indices specifically addressing SBP risk or progression remain limited. In this study, we systematically profiled HBV-related cirrhosis across disease stages and established the SBP-MI, which captured microbial alterations associated with disease progression. Furthermore, by integrating microbial and clinical parameters, we developed the SBP-RP model as a potential tool for early identification of high-risk patients and guiding preventive strategies.

Mechanistically, the microbiota–gut–liver axis provides a unifying framework for these observations. Increased intestinal permeability permits translocation of pathogen associated molecular patterns, such as lipopolysaccharide and bacterial DNA, into the portal and systemic circulation (4). This influx amplifies systemic inflammation and promotes cirrhosis associated immune dysfunction, a state of concurrent immune activation and impaired antimicrobial defenses that heightens susceptibility to infection (10). Cirrhosis is associated with mycobiome alterations, including reduced fungal diversity and increased Candida abundance. Invasive fungal infections in this population are linked to worse early outcomes, suggesting that fungal overgrowth may further weaken barrier function and increase infection risk (11). The role of fungi in SBP will be explored in future work.

In the case-control analysis, α-diversity was significantly reduced and β-diversity analyses showed a clear separation from healthy controls (**Figures 2A, B**), suggesting marked remodeling of the gut microbial community in cirrhosis complicated by SBP. Previous studies have reported that cirrhosis-associated dysbiosis worsens with decompensation and infectious episodes and is linked to greater clinical severity and adverse outcomes (12). Accordingly, the loss of diversity observed represents a microbiome feature accompanying disease progression. From a pathophysiological standpoint (4), cirrhosis is frequently associated with disrupted mucosal immune homeostasis and impaired intestinal barrier integrity. These abnormalities may act in concert with microbial community alterations to facilitate pathological bacterial translocation, thereby increasing susceptibility to SBP. In this context, a key feature was the depletion of *Prevotella, Roseburia, Faecalibacterium*, and *Bacteroides*, as shown in **Figures 2D, E**. These genera are central to microbial metabolism and the production of SCFAs (short-chain fatty acids). *Roseburia* and *Faecalibacterium* are typical butyrate producers. Butyrate not only serves as the main energy source for colonic epithelial cells but also regulates immune responses and preserves mucosal barrier integrity by upregulating tight junction proteins such as ZO-1, claudin, and occludin, thereby reducing intestinal permeability and bacterial translocation (13). Rivera-Chávez et al. showed that loss of butyrate-producing Clostridia increases luminal oxygen and drives Salmonella enterica expansion (14). By contrast, *Bacteroides* and *Prevotella* mainly produce acetate and propionate. These metabolites contribute to energy metabolism and immune regulation, and also act as substrates for butyrate-producing bacteria through cross-feeding (15), thereby indirectly supporting butyrate formation and maintaining the overall SCFAs balance. Experimental studies have demonstrated that SCFAs supplementation reduces intestinal permeability and alleviates liver injury in models of liver disease (16). Consistently, clinical studies have reported significantly decreased levels of acetate, propionate, and butyrate in patients with cirrhosis (17), suggesting that reduced SCFAs may contribute to both disease progression and microbial dysbiosis. At the same time, 16S rRNA profiling characterizes community composition rather than metabolic pathway capacity, and microbial communities often exhibit functional redundancy, whereby taxonomically distinct organisms can perform the same functions (18). Thus, compositional depletion of canonical SCFA producers does not by itself demonstrate reduced SCFA biosynthesis. Future work will incorporate shotgun metagenomics to quantify butyrate, acetate, and propionate pathways and to identify differentially abundant species, together with targeted SCFA measurements in stool. Taken together, the observed depletion of commensal genera is compatible with diminished SCFA production, impaired barrier integrity, and increased bacterial translocation risk in SBP, but direct functional assessment is warranted.

In SBP patients, we observed an increased abundance of *Streptococcus*, *Escherichia–Shigella*, *Klebsiella*, and *Veillonella* (**Figures 2D, E**). These four taxa are considered to be closely associated with the development of SBP. A significant increase in the phylum *Proteobacteria* was also detected in SBP patients. Expansion of *Proteobacteria* is regarded as a marker of microbial community instability and a potential diagnostic indicator of dysbiosis and disease risk (19). *Escherichia–Shigella* represents one of the most common pathogenic groups in SBP, with pathogenic *E. coli* shown to disrupt epithelial tight junctions and impair the mucus barrier, thereby facilitating bacterial translocation (20). *Klebsiella pneumoniae* is another frequently isolated Gram-negative bacterium in SBP ascites (21), and multidrug-resistant strains have been strongly linked to adverse outcomes (22). In recent years, the detection rate of Gram-positive *Streptococcus* has increased, suggesting its emerging role in the pathogen spectrum of SBP (23). Meanwhile, *Veillonella* was significantly enriched in the gut of SBP patients (24). As this genus contains lipopolysaccharides (LPS) in its outer membrane, it may activate the TLR4 signaling pathway, thereby increasing intestinal permeability, inducing inflammation, and accelerating SBP progression (25). Overall, the expansion of these taxa is consistent with a more pathobiont enriched community. This observation provides the rationale for SBP-MI, which summarizes the SBP associated dysbiotic profile. SBP-MI can therefore be evaluated as a tool for tracking disease progression. In addition, because SBP-MI is derived from genus-level 16S rRNA profiles, it cannot resolve species/strain-specific drivers of SBP or directly interrogate functional attributes such as virulence determinants and antimicrobial resistance. Future studies incorporating shotgun metagenomics will enable species/strain-resolved profiling and functional characterization, thereby strengthening mechanistic interpretation and clinical translation.

In the prospective follow-up, we further identified dynamic microbial changes linked to clinical outcomes in HBV-related cirrhosis with ascites. *Streptococcus* decreased, while *Bacteroidaceae*, *Prevotella*, and *Faecalibacterium* were significantly enriched (**Figure 3C**). Bert et al. (26) reported that *streptococci*, particularly *viridans group streptococci*, are common causative pathogens of SBP and related infections. Therefore, a decline in *Streptococcus* may indicate a reduced pathogenic burden, consistent with clinical improvement. In contrast, enrichment of *Bacteroidaceae*, *Prevotella*, and *Faecalibacterium* may reflect microbial rebalancing that helps maintain intestinal homeostasis and confers protective effects. In patients with disease progression, however, *Haemophilus* was significantly increased (**Figure 3E**). Although *Haemophilus* is not a typical SBP pathogen, A case report described *H. influenzae* causing severe ascitic infections (27). Its enrichment may therefore signal barrier impairment and greater risk of bacterial translocation in progressive disease. In the longitudinal analysis, changes in SBP-MI provided further insight into disease dynamics. ΔSBP-MI was significantly higher in patients with progression than in those with improvement (**Figure 3G**), suggesting that a rising SBP-MI reflects worsening dysbiosis and increased risk. By contrast, ΔHBCDI showed limited discriminatory ability. ROC analysis further highlighted the advantage of ΔSBP-MI, indicating its potential value for monitoring disease trajectories during follow-up (**Figure 3H**).

Among the 140 HBV-related cirrhosis patients with ascites followed for 6 months, 15 developed SBP. By integrating clinical and microbial indicators, we identified INR, previous history of SBP, and SBP-MI as independent predictors (**Table 2**), and on this basis constructed the SBP-RP model. These findings are broadly consistent with previous reports: elevated INR has been associated with increased susceptibility (28), and according to the EASL clinical practice guidelines (29), a prior episode of SBP substantially increases the likelihood of recurrence, underscoring the vulnerability of this subgroup. Notably, the SBP-RP model appeared to show good predictive performance, as illustrated in **Figure 4A**. Calibration analysis further demonstrated good agreement between predicted and observed risks (**Figure 4B**), indicating that the model may be both well calibrated and potentially reliable for risk prediction. This index was developed in a single-center cohort of patients with HBV-associated cirrhosis in China, and the number of incident SBP events was small. Because baseline microbiome composition, diet, clinical practice, and SBP pathogen spectra can vary by cirrhosis etiology and geography, the discrimination and calibration of SBP-MI may differ across etiologies and regions.

In conclusion, this study systematically profiled the gut microbiota of HBV-related cirrhosis across disease stages and identified SBP-MI as a candidate composite index that may reflect the imbalance between protective commensals and potentially pathogenic taxa. In prospective follow-up, SBP-MI captured microbial dynamics associated with clinical outcomes. Unlike single-taxa analyses, it integrates multiple alterations and may reflects disease stage, progression, and predictive value. Based on this, we developed the SBP-RP model by combining microbial and clinical factors, which may facilitate early identification of short-term risk patients and could inform preventive strategies. We will extend follow-up beyond six months and conduct a multicenter prospective validation to confirm the clinical utility of both SBP-MI and SBP-RP prior to wider clinical adoption.

This study has several limitations. First, strict inclusion and exclusion criteria were used to minimize medication related confounding, but this limited the sample size at our single center. The number of SBP events in the follow-up cohort was small, and follow-up was restricted to 6 months. Although we used Firth penalized logistic regression and bootstrap internal validation, the SBP-MI and SBP-RP should be regarded as exploratory, and their longer-term predictive performance will need to be assessed with extended follow-up. Second, there was no external validation cohort. Multicenter prospective studies with larger populations are needed to confirm robustness and clinical applicability, with stratified analyses for potential confounders such as age and diabetes. Third, several clinically relevant variables, including oesophago-gastric varices, portal pressure, elastography-based fibrosis indices, and ascitic protein, were not uniformly collected at baseline. Future studies will incorporate these parameters when feasible and evaluate their clinical utility. Finally, 16S rRNA sequencing provides mainly genus-level resolution and does not resolve species/strain-specific pathogens or SBP-relevant functional features, including virulence and antimicrobial resistance. Future studies using shotgun metagenomics will support species/strain-resolved taxonomy (including the mycobiome) and functional profiling.

## Data Availability

The data presented in the study are deposited in the CNGBdb repository, accession number CNP0008877.

## Glossary

SBP: Spontaneous bacterial peritonitis
HBV: Hepatitis B virus
SBP-MI: Spontaneous bacterial peritonitis microbiota-derived index
SBP-RP: Spontaneous bacterial peritonitis risk prediction
INR: International normalized ratio
AUC: Area under the curve
CDR: cirrhosis dysbiosis ratio
POD: probability of disease
HBCDI: Hepatitis B Cirrhosis Dysbiosis Indicator.
HC: Healthy controls
CC: Compensated HBV-related cirrhosis
NSBP: HBV-related cirrhosis with ascites but without SBP
TIPS: Transjugular intrahepatic portosystemic shunt
ALT: alanine aminotransferase
AST: aspartate aminotransferase
HVPG: Hepatic venous pressure gradient
LEfSe: Linear discriminant analysis effect size
OTU: Operational taxonomic unit
ROC: Receiver operating characteristic
TBil: Total bilirubin
RBC: Red blood cell
ALB: Albumin
HGB: Hemoglobin
PLT: Platelet
MELD: Model for End-Stage Liver Disease
PD: phylogenetic diversity
PCoA: Principal coordinates analysis
AFP: Alpha-fetoprotein
SCFAs: short-chain fatty acids
LPS: Lipopolysaccharide
