# Supplementary material for: From dysbiosis to prediction: a novel gut microbiota–derived index for spontaneous bacterial peritonitis in HBV-related cirrhosis
Source: Front Immunol. 2026 Feb 16;17:1753063. doi: 10.3389/fimmu.2026.1753063 (PMC12950584; doi:10.3389/fimmu.2026.1753063)
Supplement: Supplementary file 1 [file DataSheet1.docx]

**Supplementary materials**

**From dysbiosis to prediction: a novel gut microbiota–derived index for spontaneous bacterial peritonitis in HBV-related cirrhosis**

Running title: Microbiota and SBP

**Zhewen Zhou^1,^****^2†^, Xiu Sun^1,2†^, Danying Cheng^1,2^, Huichun Xing^1,2,3*^**

^1^Center of Liver Diseases Division 3, Beijing Ditan Hospital, Capital Medical University, Beijing, China

^2^National Center For Infectious Diseases, Beijing, China

^3^Peking University Ditan Teaching Hospital, Beijing, China

**^*^Correspondence:**

Huichun Xing, MD, Central of Liver Diseases Division 3, Beijing Ditan Hospital, Capital Medical University, 8 Jingshundong Street, Chaoyang District, Beijing, 100015, China.

Email: hchxing@sohu.com or [hchxing@ccmu.edu.cn](mailto:hchxing@ccmu.edu.cn), Tel: +86 10-84322291

**^†^**Zhewen Zhou and Xiu Sun have contributed equally as co-first authors.

**Supplementary Methods**

**Sample size calculation**

Sample size was estimated using published cirrhosis dysbiosis ratio (CDR) data as prior information. Bajaj et al (1). reported a stepwise decrease in the CDR across disease stages, with values of 2.05 in controls, 0.89 in compensated cirrhosis, 0.66 in decompensated cirrhosis, and 0.32 in inpatient cirrhosis. These values were used to approximate the expected effect gradient for our four-group comparison. Because the inpatient group in the Bajaj study reflected cirrhosis with infection and a clinically severe phenotype, it was used as the planning reference for the spontaneous bacterial peritonitis group. To inform within-group variability, we additionally referenced Sarangi et al.(2), who reported the CDR as mean ± SD in controls and an overall cirrhosis group (2.71 ± 1.48 and 1.55 ± 1.86, respectively). Sample-size calculations were performed in PASS using a one-way ANOVA model with equal allocation, a two-sided alpha level of 0.05, and 80% power. Two standard deviation scenarios, 1.48 and 1.86, were evaluated. Under the more stringent scenario of 1.86, the minimum required sample size was 24 participants per group, corresponding to 96 participants overall. The final retrospective dataset comprised 135 participants, including 40 healthy controls, 30 patients with compensated cirrhosis, 40 patients with decompensated cirrhosis without spontaneous bacterial peritonitis, and 25 patients with spontaneous bacterial peritonitis. All groups exceeded the minimum sample size requirement.

**Sample collection and sequencing**

Demographic and clinical data were recorded at enrollment. Fasting peripheral blood samples were collected for biochemical and virological assays. Fresh stool samples were obtained using sterile containers, transported on ice, aliquoted within 30 minutes, and stored at –80 °C until analysis. Genomic DNA was extracted from stool samples using the cetyltrimethylammonium bromide/sodium dodecyl sulfate (CTAB/SDS) method, and DNA integrity was confirmed by 1% agarose gel electrophoresis. The V3–V4 hypervariable regions of the bacterial 16S rRNA gene were amplified with barcoded primers 341F (5′-CCTAYGGGRBGCASCAG-3′) and 806R (5′-GGACTACNNGGGTATCTAAT-3′) using Phusion® High-Fidelity PCR Master Mix (New England Biolabs, USA). All PCR reactions were carried out with 15 µL of Phusion® High-Fidelity PCR Master Mix, 2 µM of forward and reverse primers, and about 10 ng template DNA. Thermal cycling consisted of initial denaturation at 98 °C for 1 min, followed by 30 cycles of denaturation at 98 °C for 10 s, annealing at 50 °C for 30 s, and elongation at 72 °C for 30 s, with a final extension at 72 °C for 5 min. PCR products were purified with the Qiagen Gel Extraction Kit (Qiagen, Germany), quantified with the Qubit® 2.0 Fluorometer (Thermo Scientific, USA), and libraries were prepared using the TruSeq® DNA PCR-Free Library Preparation Kit (Illumina, USA). Library quality was assessed with the Agilent Bioanalyzer 2100 system (Agilent Technologies, USA). Sequencing was performed on the Illumina NovaSeq platform (Illumina, USA) with paired-end reads (2 × 250 bp) on lane L1. All samples were processed in a single batch for DNA extraction, PCR amplification, and sequencing. Raw reads were demultiplexed and subjected to quality filtering before downstream analysis.

**Bioinformatics and statistical analysis**

Raw reads were quality-filtered and clustered into operational taxonomic units (OTUs) at 97% similarity. Taxonomic assignment, α-diversity and β-diversity were performed using QIIME 2 (release 2025.7), and differentially abundant taxa were identified with LEfSe. Data visualization was performed in R (v4.5.1). Normality was assessed using the Shapiro–Wilk test. Data with a normal distribution were analyzed using the Student’s t test or one-way ANOVA, whereas non-normally distributed data were analyzed using the Kruskal–Wallis or Wilcoxon rank-sum test. Trends across groups were evaluated with the Jonckheere–Terpstra test. Categorical variables were compared using the χ² test or Fisher’s exact test. Predictive modeling was performed using univariable and multivariable logistic regression. Variables with P<0.1 in univariable analysis were entered into the multivariable model, and Firth’s logistic regression was applied to reduce small-sample bias. The final model was determined after variable selection. Model discrimination was assessed by receiver operating characteristic (ROC) curve analysis, with the optimal cutoff defined by the Youden index. Internal validation was conducted using bootstrap resampling, and model calibration was evaluated by calibration intercept, slope, the Hosmer–Lemeshow goodness-of-fit test, and the Brier score. A two-sided P<0.05 was considered statistically significant.

**References**

1. Bajaj JS, Heuman DM, Hylemon PB, Sanyal AJ, White MB, Monteith P, et al. Altered Profile of Human Gut Microbiome Is Associated with Cirrhosis and Its Complications. *J Hepatol* (2014) 60(5):940-7. Epub 2014/01/01. doi: 10.1016/j.jhep.2013.12.019.

2. Sarangi AN, Goel A, Singh A, Sasi A, Aggarwal R. Faecal Bacterial Microbiota in Patients with Cirrhosis and the Effect of Lactulose Administration. *BMC Gastroenterol* (2017) 17(1):125. Epub 2017/11/29. doi: 10.1186/s12876-017-0683-9.

**Supplementary Tables**

| **Table S1. Clinical parameter changes in improved ascites patients** | | | |
| --- | --- | --- | --- |
| Characteristics | Improved group  Baseline (n=32) | Improved group Follow-up (n=32) | p value |
| ALT (U/L) | 29.00 (16.82, 38.40) | 22.00 (14.83, 33.30) | 0.192 |
| AST (U/L) | 31.25 (25.53, 52.70) | 32.10 (22.90, 39.80) | 0.161 |
| ALB (g/L) | 34.50 (29.95, 39.25) | 36.40 (32.05, 44.12) | 0.028 |
| TBil (μmol/L) | 27.20 (15.35, 52.88) | 22.80 (16.60, 31.75) | 0.016 |
| INR | 1.29 (1.14, 1.49) | 1.24 (1.14, 1.44) | 0.220 |
| WBC (109/L) | 3.35 (2.46, 4.38) | 3.33 (2.81, 4.30) | 0.922 |
| RBC (1012/L) | 3.88 (3.19, 4.21) | 4.01 (3.73, 4.34) | 0.018 |
| HGB (g/L) | 113.92 ± 24.54 | 124.69 ± 22.56 | 0.003 |
| PLT (109/L) | 65.06 ± 37.31 | 70.24 ± 39.93 | 0.127 |
| Cr (μmol/L) | 71.14 ± 16.29 | 71.47 ± 17.91 | 0.773 |
| AFP (ng/mL) | 4.55 (2.28, 9.54) | 3.83 (1.93, 6.49) | 0.006 |
| Log10HBV DNA (IU/mL) | 1.79 (1.00, 4.34) | 1.18 (1.00, 1.18) | <0.001 |
| MELD | 11.00 (9.00, 14.25) | 10.50 (8.75, 13.00) | 0.022 |

Note: Data were shown as mean ± SD, median (IQR).

Abbreviations: ALT, alanine aminotransferase; AST, aspartate aminotransferase; ALB, albumin; TBil, total bilirubin; INR, international normalized ratio; WBC, white blood cell ; RBC, red blood cell ; HGB, hemoglobin; PLT, platelet ; Cr, creatinine; AFP, alpha-fetoprotein; HBV DNA, hepatitis B virus DNA; MELD, model for end-stage liver disease.

**Table S2. Clinical parameter changes in progressed ascites patients**

| Characteristics | Progression group  Baseline (n=8) | Progression group  Follow-up (n=8) | p value |
| --- | --- | --- | --- |
| ALT (U/L) | 26.14 ± 9.63 | 32.69 ± 16.79 | 0.123 |
| AST (U/L) | 27.51 ± 9.40 | 32.38 ± 15.55 | 0.148 |
| ALB (g/L) | 33.44 ± 8.46 | 30.74 ± 4.81 | 0.173 |
| TBil (μmol/L) | 23.93 ± 14.11 | 30.44 ± 20.17 | 0.036 |
| INR | 1.25 (1.09, 1.66) | 1.43 (1.22, 1.70) | 0.293 |
| WBC (10^9^/L) | 2.52 (2.26, 3.09) | 2.21 (1.95, 4.14) | 0.441 |
| RBC (10^12^/L) | 3.46 ± 0.90 | 3.39 ± 0.63 | 0.639 |
| HGB (g/L) | 99.38 ± 22.20 | 104.90 ± 17.18 | 0.199 |
| PLT (10^9^/L) | 91.53 ± 108.28 | 90.55 ± 102.77 | 0.730 |
| Cr (μmol/L) | 74.50 ± 20.77 | 77.01 ± 32.20 | 0.635 |
| AFP (ng/mL) | 1.57 ± 0.56 | 1.31 ± 0.64 | 0.118 |
| Log10HBV DNA (IU/mL) | 1.00 (1.00, 1.00) | 1.00 (1.00, 1.18) | 0.149 |
| MELD | 11.88 ± 5.59 | 13.75 ± 5.95 | 0.006 |

Note: Data were shown as mean ± SD, median (IQR).

Abbreviations: ALT, alanine aminotransferase; AST, aspartate aminotransferase; ALB, albumin; TBil, total bilirubin; INR, international normalized ratio; WBC, white blood cell ; RBC, red blood cell ; HGB, hemoglobin; PLT, platelet ; Cr, creatinine; AFP, alpha-fetoprotein; HBV DNA, hepatitis B virus DNA; MELD, model for end-stage liver disease.

**Table S3. Baseline clinical characteristics of HBV-related cirrhosis with ascites patients**

| Characteristics | HBV-related cirrhosis with ascites (n=140) |
| --- | --- |
| Male (%) | 98 (70.0%) |
| Age | 53.5 ± 12.3 |
| ALT (U/L) | 24.5 (14.3–36.6) |
| AST (U/L) | 31.4 (23.9–49.5) |
| ALB (g/L) | 32.7 ± 6.5 |
| CHE (U/L) | 3099.5 (2263.2–4594.2) |
| NE (%) | 58.5 (50.4–68.2) |
| WBC (10^9^/L) | 3.0 (2.1–4.3) |
| RBC (10^12^/L) | 3.6 ± 0.7 |
| PLT (10^9^/L) | 59.1 (41.0–83.2) |
| INR | 1.4 (1.2–1.6) |
| TBil (µmol/L) | 26.8 (15.4–48.0) |
| Cr (µmol/L) | 68.7 (58.4–80.1) |
| log10HBV DNA (IU/mL) | 1.2 (1.0–3.6) |
| Na (mmol/L) | 140.7 (139.0–142.5) |
| GLU (mmol/L) | 5.2 (4.6–6.2) |
| Diabetes (%) | 30 (21.4%) |
| Previous history of SBP (%) | 35 (25.0%) |

Note: Data were shown as mean ± SD, median (IQR), or n (%).

Abbreviations: HBV, hepatitis B virus; ALT, alanine aminotransferase; AST, aspartate aminotransferase; ALB, albumin; CHE, cholinesterase; NE, neutrophil percentage; WBC, white blood cell count; RBC, red blood cell count; PLT, platelet count; INR, international normalized ratio; TBil, total bilirubin; Cr, creatinine; GLU, glucose; SBP, spontaneous bacterial peritonitis.

| **Table S4. Association between previous history of SBP and development of SBP during follow-up** | | | |
| --- | --- | --- | --- |
| Characteristics | Developed SBP  during follow-up | No SBP  during follow-up | Total |
| Previous history of SBP | 7 (20.0%) | 28 (80.0%) | 35 |
| No previous history of SBP | 8 (7.6%) | 97 (92.4%) | 105 |
| Total | 15 | 125 | 140 |

Note: Values are n (row %).

Abbreviations: SBP, spontaneous bacterial peritonitis.

**Supplementary Figures**

**Figure S1.**

**
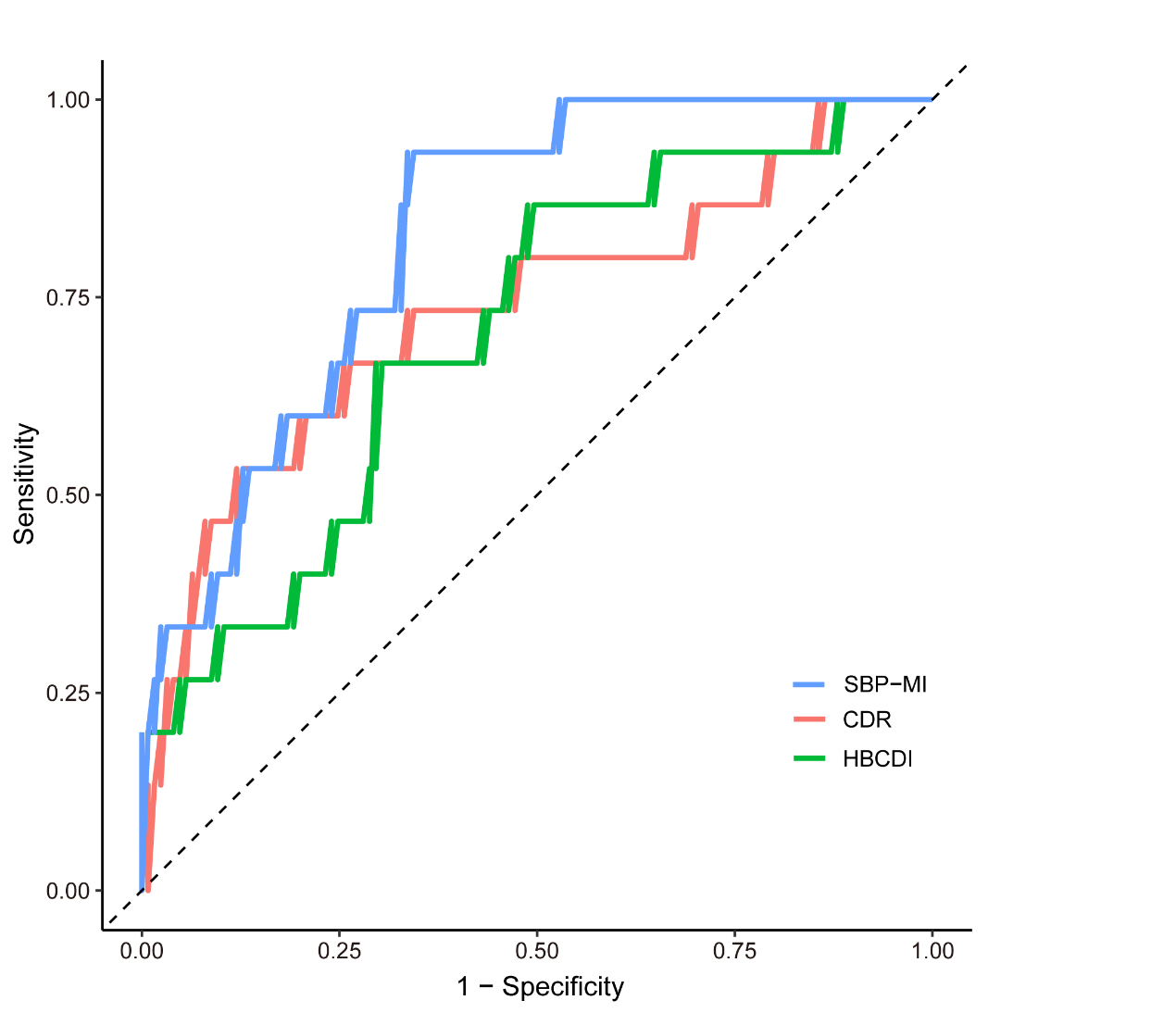
**

**Fig S1. ROC curves for predicting SBP using SBP-MI, CDR, and HBCDI.**

Abbreviations: ROC, receiver operating characteristic; SBP, spontaneous bacterial peritonitis; SBP-MI: Spontaneous bacterial peritonitis microbiota-derived index; CDR: cirrhosis dysbiosis ratio**;** HBCDI: Hepatitis B Cirrhosis Dysbiosis Indicator.
